# Shift Work and Serum Vitamin D Levels: A Systematic Review and Meta-Analysis

**DOI:** 10.3390/ijerph19158919

**Published:** 2022-07-22

**Authors:** Margherita Martelli, Gianmaria Salvio, Lory Santarelli, Massimo Bracci

**Affiliations:** 1 Occupational Health, Department of Clinical and Molecular Sciences, Polytechnic University of Marche, 60126 Ancona, Italy; m.martelli@pm.univpm.it; 2 Endocrinology Clinic, Department of Clinical and Molecular Sciences, Polytechnic University of Marche, 60126 Ancona, Italy; g.salvio@pm.univpm.it

**Keywords:** vitamin D, shift work, night work, circadian rhythm, workers, job

## Abstract

Vitamin D deficiency and insufficiency are highly prevalent conditions worldwide due to several factors, including poor sun exposure. Shift workers may be exposed to the risk of hypovitaminosis D due to fewer opportunities for sunlight exposure compared to day workers. A systematic review of the PubMed, SCOPUS, and EMBASE databases was conducted according to the Preferred Reporting Items for Systemic Reviews and Meta-Analyses (PRISMA) statement to investigate the effect of shift work on vitamin D levels. Mean differences (MD) and 95% confidence intervals (CI) of serum 25-OH-D levels in shift workers and non-shift workers were calculated. A total of 13 cross-sectional studies were included in the meta-analysis. We found significantly lower levels of serum 25-OH-D in shift workers compared with non-shift workers (MD: −1.85, 95% CI [−2.49 to −1.21]). Heterogeneity among included studies was high (I^2^ = 89%, *p* < 0.0001), and neither subgroup analysis nor meta-regression were able to identify specific sources of the heterogeneity that may be related to the different characteristics of shift work among studies. The monitoring of serum vitamin D levels and prompt correction of any deficiencies should be considered in shift workers. Notably, since a large part of the observations are derived from Koreans, larger epidemiological studies are needed in other populations.

## 1. Introduction

As shown by worldwide data, vitamin D deficiency (25-OH-D levels below 20 ng/mL) and vitamin D insufficiency (25-OH-D levels between 21 and 29 ng/mL) are serious global health problems [1,2]. Indeed, approximately one billion people are estimated to be affected by vitamin D insufficiency [3]. The prevalence of this condition varies widely by geographic area, latitude, lifestyle habits (work, sun exposure, type of clothing used), dietary habits, gender, and genetic factors [4]. Notably, since the optimal 25-OH-D levels are still debated, the above-mentioned definitions of hypovitaminosis D are not universally accepted. Consequently, maintaining 25-OH-D levels > 20 ng/mL is widely accepted for most of the population, but individual objectives may vary [5].

Approximately 90% of the vitamin D detectable in human serum is synthesized in the skin from a cholesterol-like precursor (7-dehydrocholesterol) present in epidermal cells. This, as a result of exposure to ultraviolet B (UV-B) radiation from sunlight, is converted to vitamin D3 (cholecalciferol). The remaining portion (around 10%) is obtained from foods such as oily fish, egg yolk, animal liver, and some types of mushrooms. Animal sources contain vitamin D3, while plant sources contain vitamin D2 (ergocalciferol). Vitamin D2 and vitamin D3 are both biologically inactive and they undergo further enzymatic conversions: first, they undergo a 25-hydroxylation in the liver, becoming 25-(OH)-D (calcifediol), the main circulating form of vitamin D, with a half-life of 2–3 weeks. Then, 25-(OH)-D is finally activated by 1-alpha-hydroxylation in the kidney, becoming 1,25(OH)2D (calcitriol) with a half-life of 4–6 h [6,7]. Vitamin D plays a key role in the homeostasis of calcium metabolism and in the process of bone modeling and remodeling [4]; therefore, deficiency of this vitamin is associated with osteoporosis and a higher incidence of fractures [6,8].

Since endogenous vitamin D synthesis is highly dependent on sunlight, poor exposure to solar radiation is the leading cause of hypovitaminosis D [9]. A systematic review of 71 studies conducted worldwide found that occupation is a major factor impacting vitamin D levels and that indoor workers are at greater risk of developing hypovitaminosis D than outdoor workers [10]. In addition, several studies have shown an association between low vitamin D levels and many other diseases, such as autoimmune disorders, cardiovascular disease, infectious diseases, type 2 diabetes mellitus, cancer, and neurological and neuropsychiatric disorders, such as schizophrenia, dementia, and depression [6,11]. Shift work refers to a work organization in which the workers have shift schedules to cover more than the usual 8 h day, up to and including the whole 24 h [12]. Shift work—in particular, shift work including night shifts—may be a risk factor for vitamin D deficiency, since shift workers are likely to have fewer opportunities for sunlight exposure than day workers [13]. In addition, shift workers are more likely to consume unhealthy foods, as well as displaying a poor tendency to take vitamin supplements [14,15].

Several studies have shown an association between shift work and overweight [16,17,18,19]. At the same time, it is known that hypovitaminosis D is common in obese individuals and that BMI and fat mass are factors inversely related to 25-OH-D levels [1,20,21].

Since several factors associated with shift work can predispose shift workers to lower vitamin D levels, the objective of the present meta-analysis is to determine whether shift work constitutes a risk of low levels of vitamin D.

## 2. Materials and Methods

### 2.1. Search Strategy

A systematic search was conducted through the Scopus, PubMed, and EMBASE databases from January to February 2022. The terms “vitamin D”, “25-hydroxyvitamin-D”, or “25-OH-D” were combined with “shift work”, “night work”, “night shift work”, “shiftwork”, “shift schedule”, or “indoor work”. In particular, the following query strings were used: “(vitamin D* OR 25-Hydroxyvitamin-D OR 25(OH)D OR 25-OH-D) AND (shift work* OR night work* OR night shift work* OR shiftwork* OR nightwork* OR shift schedule OR indoor work*)” (PubMed, EMBASE), and “TITLE-ABS-KEY ((vitamin D* OR 25-Hydroxyvitamin-D) AND (shift work* OR night work* OR night shift work* OR shiftwork * OR nightwork* OR shift schedule OR indoor work*))” (Scopus). The search was conducted by two authors (M.M. and G.S.) according to the guidelines of the Preferred Reporting Items for Systematic Reviews and Meta-Analyses (PRISMA) statement [22]. The study was registered on PROSPERO (CRD42022341088).

### 2.2. Selection Criteria

The eligible studies were selected following the PICO model: Population (adult workers), Intervention (shift work), Comparison (non-shift work), Outcome (25-OH-D serum levels). All the studies reporting vitamin D levels in adult workers (differentiated in shift workers and non-shift workers) until 2021 were included. In vitro or animal studies, case reports, and non-English papers were excluded.

### 2.3. Data Extraction and Quality Assessment

Three authors (M.M., G.S., and M.B.) performed data extraction. The following data were collected: first author, year, study design, method of vitamin D measurement, number of subjects, gender, age, body mass index (BMI), mean serum 25-OH-D levels. When the values of 25(OH)D levels were reported in nmol/L, they were converted into ng/mL by dividing them by 2.496. When the standard error of the mean (SEM) was reported, standard deviation (SD) was calculated by multiplying SEM by the square root of the number of subjects. The quality of evidence (QoE) was assessed by one researcher (M.M.) using the Cambridge Quality Checklists (CQCs) [23]. In brief, the CQCs were designed to identify high-quality studies for systematic reviews and meta-analyses and provide information on correlations (five items investigating sampling method, response and retention rates, sample size, and correlates/outcome assessment), risk factors (definition of study design), and causal risk factors (how the risk factor is causally related to the outcome).

### 2.4. Statistical Analysis

The analysis was performed using RevMan software v. 5.4 (Cochrane Collaboration, Oxford, UK) and Comprehensive Meta-Analysis v. 3 (Biostat Inc., Englewood, NJ, USA). Mean difference and 95% confidence interval (CI) were calculated to compare serum vitamin D levels between shift workers and non-shift workers, and meta-analysis was performed using a random-effect model. The I^2^ statistic was applied to inspect heterogeneity, with I^2^ > 50% and *p* < 0.1 indicating high between-study heterogeneity. Publication bias was assessed by funnel plot asymmetry as well as Egger’s test. To investigate the source of heterogeneity, subgroup analysis (based on methods of vitamin D measurement and gender prevalence), meta-regression (with adjustment for age and BMI), and sensitivity analysis (omitting each single study to explore its effect on the overall meta-analysis) were conducted. Statistical significance was set at 0.05.

## 3. Results

### 3.1. Study Selection

**Figure 1:**
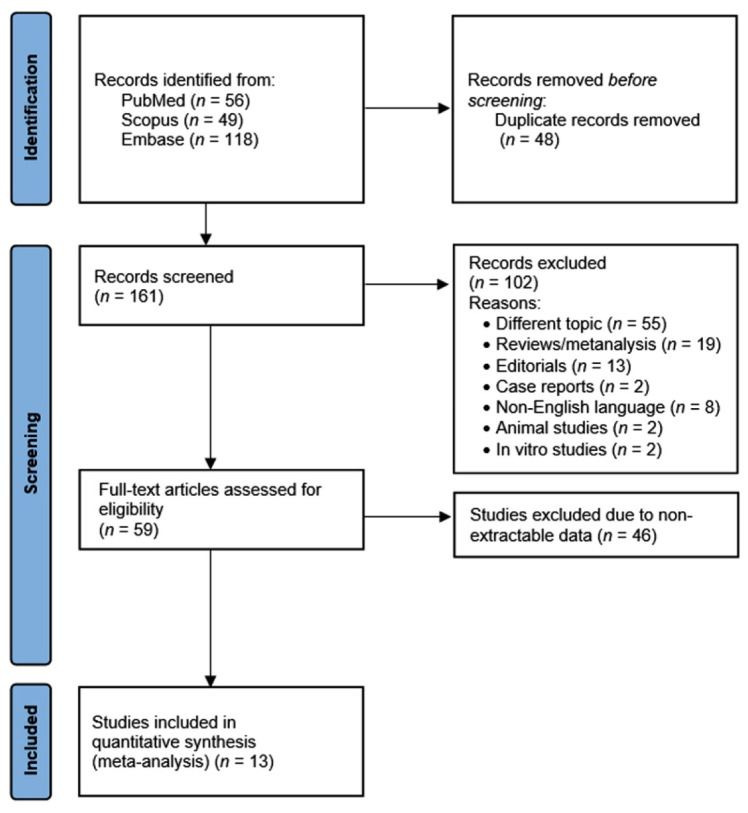
Preferred Reporting Items for Systematic Review and Meta-Analysis Protocols (PRISMA-P) flowchart.

Using the above-mentioned search strategy, 223 abstracts were extracted. After the removal of 48 duplicates, 161 articles were screened. Of these, 102 were identified by title or abstract as papers on other topics, review articles, editorials, case reports, animal or in vitro studies, or non-English articles. Of the remaining 59 full-text articles assessed for eligibility, 46 were excluded due to non-extractable data (e.g., vitamin D values not specified, not clearly distinguished between shift and non-shift workers, or data summarized with different descriptive statistics to mean and SEM or SD). Finally, 13 studies [12,24,25,26,27,28,29,30,31,32,33,34,35] were included in the present meta-analysis (Figure 1). All the included studies had a cross-sectional design, and their main characteristics are presented in Table 1. The characteristics of the workers and shift work in the included studies are summarized in Table 2. The results of QoE are shown in Table 3.

### 3.2. Differences in Vitamin D Levels between Shift Workers and Non-Shift Workers

**Figure 2:**
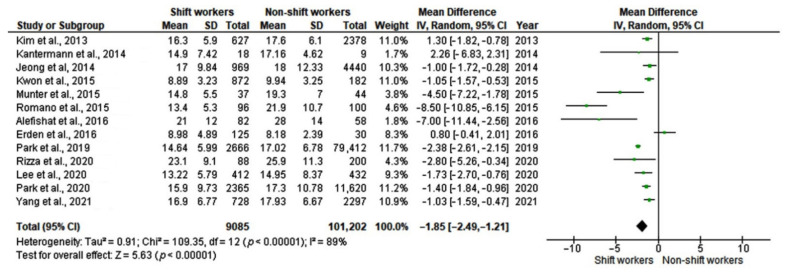
Forest plot showing mean differences in serum 25-hydroxyvitamin D (25-OH-D) levels (ng/mL) in shift workers and non-shift workers [12,24,25,26,27,28,29,30,31,32,33,34,35].

**Figure 3:**
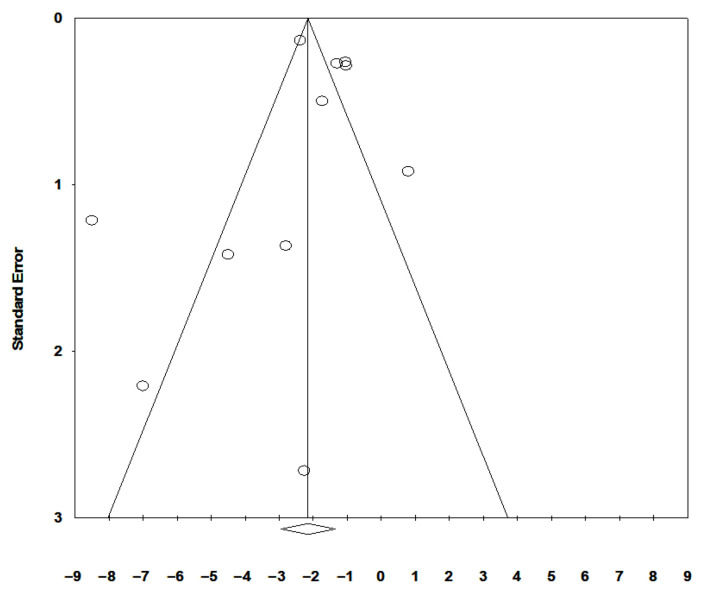
Funnel plot of the included studies showing no significant asymmetry.

Based on 13 studies including a total of 110,287 subjects, a random-effects model revealed significantly lower serum 25-OH-vitamin D levels in shift workers compared with non-shift workers (mean difference, MD: −1.85, 95% CI [−2.49 to −1.21]) with high heterogeneity between studies (I^2^ = 89%, *p* < 0.0001) (Figure 2). Egger’s test (*p* = 0.37) and visual examination of funnel plots (Figure 3) indicated no significant publication bias over the included studies. As far as sensitivity analysis is concerned, omitting the study of Park et al. [30], a slight decrease in between-study heterogeneity was observed (I^2^ = 83%, *p* < 0.0001), without substantial changes in the estimate of the effect size (MD: −1.75, 95% CI [−2.42 to −1.09]). Some studies reported 25-OH-D mean values with high SD. This suggests that a non-normal distribution should be carefully considered in studies on 25-OH-D serum levels.

It would have been interesting to evaluate the prevalence of people with insufficient vitamin D levels among shift and non-shift workers. However, the selected studies rarely reported these data and different cut-offs were used, making the data not analyzable. The type of shift work and particularly the number of night shifts worked per month are likely high related to sunlight exposure and therefore to vitamin D levels. The investigation of this relation was not possible due to the lack of a clear definition of “shift work” in several studies.

### 3.3. Differences in Vitamin D Levels between Shift Workers and Non-Shift Workers—Subgroup Analysis

#### 3.3.1. Methods of Measurement

**Figure 4:**
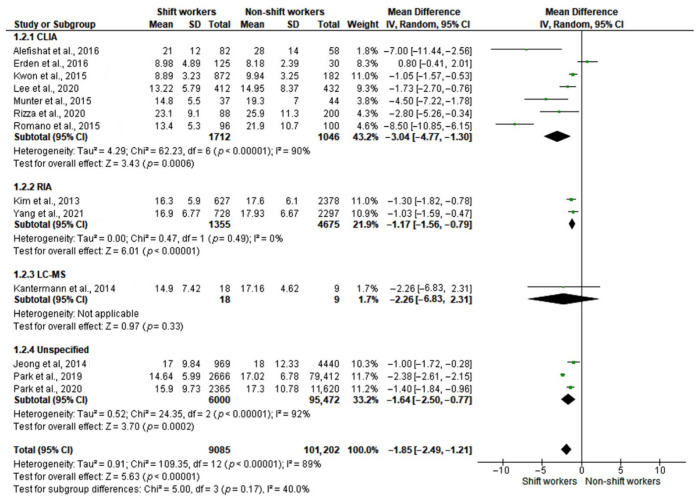
Forest plots showing mean differences in serum 25-hydroxyvitamin D (25-OH-D) levels (ng/mL) in shift workers and non-shift workers (subgroup 1: methods of vitamin D measurement) [12,24,25,26,27,28,29,30,31,32,33,34,35].

In order to explore the source of heterogeneity, a subgroup analysis according to the methods of serum 25-OH-D level measurement was performed. The test for subgroup differences indicated that there was no statistically significant subgroup effect (*p* = 0.17), suggesting that the method of measurement does not modify the effect of shift work on serum vitamin D levels. However, most of studies reported vitamin D levels measured by chemiluminescent immunoassay (CLIA), whereas other methods were used in a smaller number of studies (Figure 4). In addition, in three large studies [26,28,30], the method of measurement was not specified.

**Figure 5:**
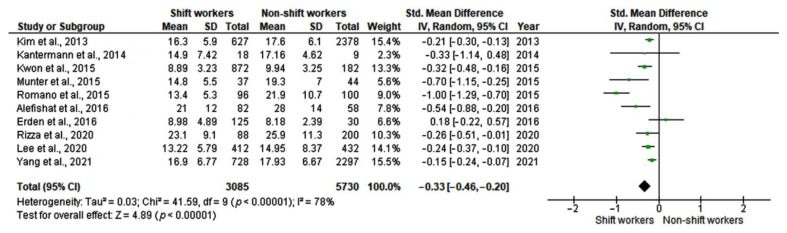
Forest plots showing mean differences in serum 25-hydroxyvitamin D (25-OH-D) levels (ng/mL) in shift workers and non-shift workers (omitting unspecified methods of vitamin D measurement) [12,24,25,27,29,31,32,33,34,35].

Omitting these three studies, the analysis was still significant, with a slight reduction in heterogeneity (MD: −0.33, 95% CI [−0.45 to −0.20]; I^2^ = 78%, *p* < 0.0001) (Figure 5).

#### 3.3.2. Sex Differences

**Figure 6:**
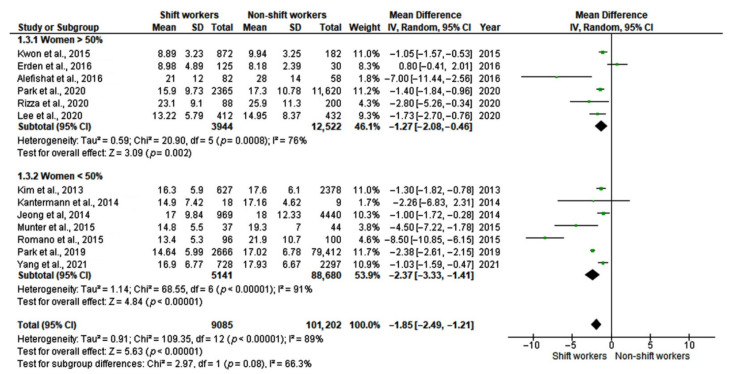
Forest plots showing mean differences in serum 25-hydroxyvitamin D (25-OH-D) levels (ng/mL) in shift workers and non-shift workers (subgroup 2: gender prevalence) [12,24,25,26,27,28,29,30,31,32,33,34,35].

The subgroup analysis showed a lower effect of shift work on serum vitamin D levels in studies where female workers were >50%, with lower heterogeneity (MD: −1.27, 95% CI [−2.08 to −0.46]; I^2^ = 76%, *p* = 0.0008) compared with studies where women were < 50% (MD: −2.37, 95% CI [−3.33 to −1.41]; I^2^ = 91%, *p* < 0.0001), but the subgroup effect was not statistically significant (*p* = 0.08) (Figure 6). However, a far smaller number of subjects contributed to the female prevalence subgroup (16,466 vs. 93,821), and the analysis may not have been able to detect subgroup differences.

#### 3.3.3. Meta-Regression Analysis

**Figure 7:**
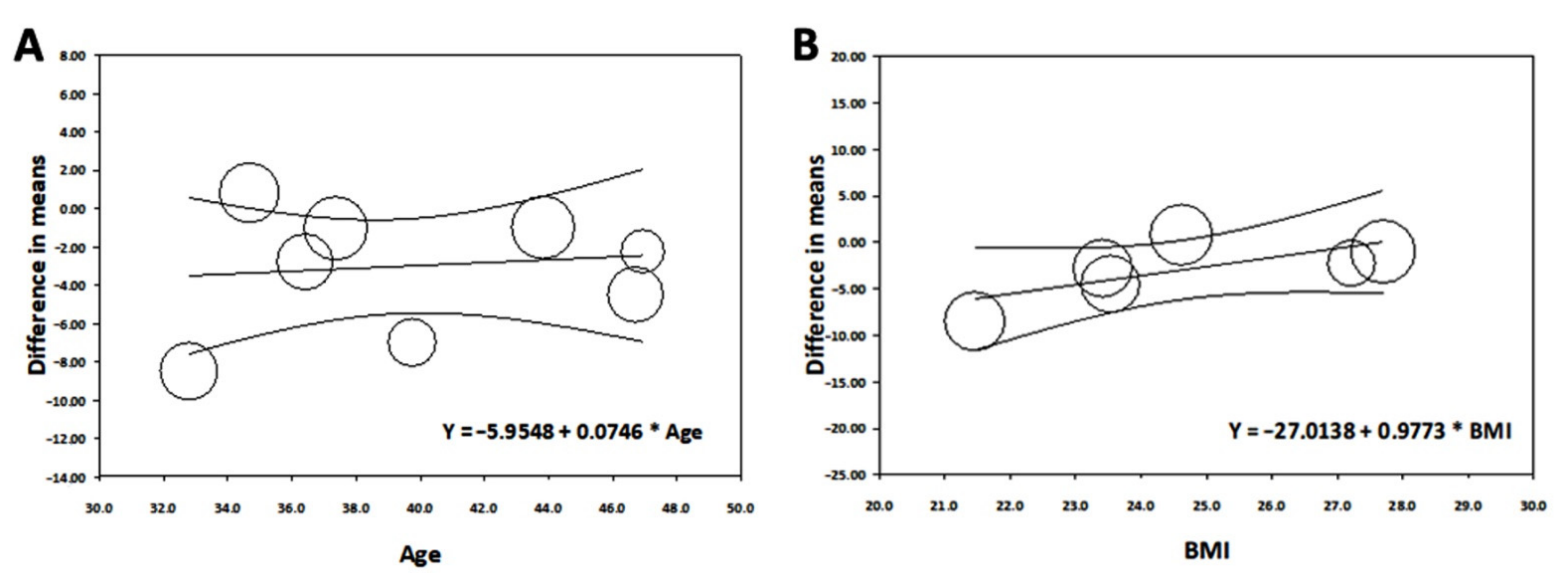
Meta-regression analysis performed for mean differences in serum vitamin D levels between shift and non-shift workers, with age (**A**) and BMI (**B**) as covariates.

Among the included studies, data on mean age and mean BMI were provided in eight [12,24,25,27,29,30,32,33] and six papers [12,25,27,29,32,33], respectively. Random-effect meta-regression analysis did not show any relationship between age (β = 0.075; 95% CI [−0.323 to 0.472]; *p* = 0.7) or BMI (β = 0.977; 95% CI [−0.187 to 2.141]; *p* = 0.1) and mean differences in vitamin D levels (Figure 7).

## 4. Discussion

The present metanalysis shows significantly lower serum vitamin D levels in shift workers compared with non-shift workers. The main cause of this result may be that shift workers are exposed to lower levels of sunlight than other categories of workers [12]. In addition, the tendency of shift workers to eat meals at irregular times and their tendency to eat a diet rich in fatty and junk food could lead to a reduced dietary intake of vitamin D [13,14]. It is also known that shift workers tend to have a BMI higher than the general population [15,36,37], a factor that could lead to the increased sequestration of vitamin D in adipose tissue and, consequently, lower values of circulating vitamin D [38,39].

In shift workers, the health risks posed by vitamin D deficiency can be combined with the risks of altered circadian rhythms. The expression of vitamin D receptors in areas of the brain that regulate the sleep–wake cycle has been shown [40]. Some studies highlighted an association between vitamin D deficiency and sleep disturbances [41,42,43]. Sleep disorders commonly afflict shift workers; in fact, we can speak of Shift Work Disorder, a sleep–wake cycle disorder characterized by symptoms such as insomnia and excessive sleepiness, due to the deregulation of the circadian rhythm that affects this category of workers [44,45].

Hypovitaminosis D has been extensively studied in bone metabolism disorders and particularly in osteoporosis [2]. Fracture risk in shift workers has never been evaluated in the past, but a recent study by Bukowska-Damska et al. showed a higher rate of bone turnover in female night shift workers, suggesting a potential link between osteoporosis and shift work [46]. Prospective longitudinal studies instead of cross-sectional studies may be more appropriate in assessing the incidence of fractures in shift workers. Accordingly, shift workers should be followed over time for vitamin D deficiency and fracture risk in periodic health surveillance.

Vitamin D deficiency has also been correlated with the occurrence of cardiovascular disease. In particular, several meta-analyses have demonstrated an inverse correlation between vitamin D concentration and cardiovascular mortality [47,48,49]. In addition, vitamin D deficiency has been associated with reduced HDL levels and increased LDL levels [50]. In shift workers, the low serum levels of vitamin D could contribute to the increased cardiovascular risk found in this category of workers [45]. This should be taken into account in the health surveillance of these workers.

Vitamin D metabolism involves several biochemical reactions and metabolites; 25(OH)D is the most represented in the bloodstream due to its long half-life and thus represents the gold standard for estimating the body’s reserves of vitamin D [51]. Different assays for the measurement of 25-OH-D levels are available and this may constitute a possible source of heterogeneity among studies. A subgroup analysis was performed to investigate whether the 25-OH-D measurement method influenced the results of the meta-analysis. However, in three studies [26,28,30], two of which were very large [28,30], the measurement methods were not reported. In addition, CLIA was the method of choice in most of the studies, whereas alternative techniques, including radioimmunoassay (RIA) and liquid chromatography–mass spectrometry (LC-MS), had been used only in three studies [24,25,27]. This is in line with current clinical practice. Indeed, immunoassays (IAs) are currently the method of choice for vitamin D measurement in most laboratories, given their automation and rapidity. However, cross-reactivity between similar vitamin D metabolites is the main drawback of these methods, whose specificity is strictly dependent on the quality of the antibody used [52]. Indeed, despite relatively low intralaboratory variability [53], a recent interlaboratory comparison study showed an acceptable coefficient of variation (lower than 10%) in only 50% of IAs, differently from the LC-MS assays, which provided comparable results in most cases [54]. As evidence of this, removal of studies that had used measurement methods other than IAs did not lead to a reduction in heterogeneity in our meta-analysis.

In the present meta-analysis, a lower effect of shift work on low levels of vitamin D was seen in female-dominated studies. In the literature, there is no consensus on how gender affects vitamin D levels; in fact, some studies report higher average levels in males, and some others in females [55,56,57]. These aspects should be explored further in the future, taking into account possible gender-related bias, such as increased awareness of osteoporosis in women, which could lead to more frequent vitamin D supplementation in female workers.

Ageing is a well-established risk factor for hypovitaminosis D [58] and several epidemiological studies have shown that the prevalence of hypovitaminosis D increases linearly with BMI, with lower vitamin D in overweight and obese subjects [59]. In order to explore whether age or BMI could modify the effect size of shift work on serum vitamin D levels, we performed a meta-regression including age and BMI as covariates. Surprisingly, neither age nor BMI seems to affect vitamin D levels in shift workers. This may be related to the specific population examined, including a working population with a limited age range (18–65 years) and subjects with BMI values contained within the limits of normal weight or slight overweight.

The present meta-analysis has the strength that it is currently the only meta-analysis investigating vitamin D levels in shift workers. In addition, strict adherence to validated investigation methods (PICO criteria and Cambridge Quality Checklists) ensures the transparency and reproducibility of the results. However, our study also presents some limitations. First, all the included studies had a cross-sectional design with low–moderate quality. Second, interstudy heterogeneity was high, and neither subgroup analysis nor meta-regression were able to identify specific sources of the heterogeneity. Several definitions of “shift work” were used in the studies analyzed in this meta-analysis. This is a known problem of epidemiological studies investigating the association between shift work and cancer, and it led the IARC to convene a working group to establish a uniform definition of shift and night work [12]. Similarly, the problem concerns the studies analyzing the relation between shift work and vitamin D levels. The same recommendations of the IARC working group report should be extended in future studies about shift workers and vitamin D levels [12]. Since the displacement from the solar day caused by shift schedules (particularly during non-day shifts) may have a strong influence on sunlight exposure and consequently on vitamin D levels, the different characteristics of shift work may be the cause of the high interstudy heterogeneity observed. Third, most of the studies included random evaluations of serum vitamin D levels, not considering the season. Since vitamin D levels are higher in the spring–summer and lower in the fall–winter periods, some of the heterogeneity could depend on this factor, which needs clarification. Finally, since most of the subjects studied (96.5%) were Korean, the generalizability of our observations may be questionable and larger epidemiological studies are needed in other populations.

## 5. Conclusions

Our study is the first meta-analysis showing that shift workers have lower levels of vitamin D compared with non-shift workers. Interstudy heterogeneity was high, not explained by age, sex, BMI, and methods of serum 25-OH-D level measurement. It is likely that the characteristics of the shift work, particularly the number of nights worked per month, play a critical role. According to our results, the monitoring of vitamin D levels and the prompt correction of deficiencies to prevent fracture risk should be considered in the periodic health surveillance of this category of workers. It should be noted that since a large part of the observations derive from Koreans, the generalizability of our observations may be questionable and larger epidemiological studies are needed in other populations.

## Figures and Tables

**Table 1:** Main characteristics of the included studies.

			Shift Workers				Non-Shift Workers				
Authors, Year	Country	Vitamin D Assay	Number	Age (Years)	BMI (kg/m^2^)	Vitamin D Levels (ng/mL)	Number	Age (Years)	BMI (kg/m^2^)	Vitamin D Levels (ng/mL)	Gender Prevalence
Yang et al., 2021 [24]	U.S.A.	RIA	728	30.09 ± 14.18	-	16.90 ± 6.77	2297	39.67 ± 14.50	-	17.93 ± 6.67	Men
Park et al., 2020 [28]	Korea	-	2365	-	-	15.90 ± 9.73	11,620	-	-	17.30 ± 10.78	Women
Rizza et al., 2020 [29]	Italy	CLIA	88	44.70 ± 7.90	24.30 ± 5.40	23.10 ± 9.10	200	47.60 ± 9.80	23.20 ± 4.00	25.90 ± 11.30	Women
Lee et al., 2020 [12]	Korea	CLIA	412	29.02 ± 6.99	21.08 ± 2.79	13.22 ± 5.79	432	36.39 ± 11.38	21.83 ± 2.82	14.95 ± 8.37	Women
Park et al., 2019 [30]	Korea	-	2666	35.84 ± 6.31	-	14.64 ± 5.99	79,412	39.88 ± 6.23	-	17.02 ± 6.78	Men
Alefishat et al., 2016 [31]	Jordan	CLIA	82	-	-	21.00 ± 12.00	58	-	-	28.00 ± 14.00	Women
Erden et al., 2016 [32]	Turkey	CLIA	125	35.06 ± 9.60	24.46 ± 3.84	8.98 ± 4.89	30	34.30 ± 7.18	24.78 ± 2.68	8.18 ± 2.39	Women
Romano et al., 2015 [33]	Italy	CLIA	96	42.50 ± 7.60	26.40 ± 3.60	13.40 ± 5.30	100	51.20 ± 13.00	28.00 ± 2.20	21.90 ± 10.70	Men
Munter et al., 2015 [34]	Israel	CLIA	37	-	-	14.80 ± 5.50	44	-	-	19.30 ± 7.00	Men
Kwon et al., 2015 [35]	Korea	CLIA	872	-	-	8.89 ± 3.23	182	-	-	9.94 ± 3.25	Women
Kantermann et al., 2014 [25]	Belgium	LC-MS	18	43.40 ± 6.20	26.90 ± 4.90	14.90 ± 7.42	9	44.70 ± 4.80	29.30 ± 4.70	17.16 ± 4.62	Men
Jeong et al., 2014 [26]	Korea	-	969	-	-	17.00 ± 9.84	4440	-	-	18.00 ± 12.33	Men
Kim et al., 2013 [27]	Korea	RIA	627	33.80 ± 9.50	23.10 ± 3.50	16.30 ± 5.90	2378	37.10 ± 8.50	23.50 ± 3.40	17.60 ± 6.10	Men

BMI = body mass index; CLIA = chemiluminescence immunoassay; LC-MS = liquid chromatography tandem mass spectrometry; RIA = radioimmunoassay; continuous variables are presented as mean ± standard deviation.

**Table 2:** Characteristics of workers and shift work.

Authors, Year	Type of Workers (and Comparison)	Definition of Shift Work	Outdoor/Indoor Work
Yang et al., 2021 [24]	Unspecified—National survey	Working regular evening shifts, regular night shifts, and/or rotating shifts	Unspecified
Park et al., 2020 [28]	Unspecified—National survey	Working night shifts or rotating shift	Unspecified
Rizza et al., 2020 [29]	Hospital workers	Shift schedule of four to seven 12 h nights per month, followed by 2 days off	Indoor
Lee et al., 2020 [12]	Hospital workers	≥6 night shifts (working hours of 6:00 p.m. to 8:00 a.m., 7:00 p.m. to 7:00 a.m., or 10:00 p.m. to 7:00 a.m.) in a month	Indoor
Park et al., 2019 [30]	Unspecified	Participants who responded to the question “In the past year, during which time of the day have you worked the most?” using the option “I work during other hours” rather than “I work mostly during the day (between 6 a.m. and 6 p.m.)”	Unspecified
Alefishat et al., 2016 [31]	Employees	Subjects working from 4:00 p.m. till 7:00 a.m. at least 4 times per month for at least 3 years	Unspecified
Erden et al., 2016 [32]	Anesthesia personnel (versus office workers)	Night shifts (unspecified)	Indoor
Romano et al., 2015 [33]	Factory workers	2 or 3 night shifts per week	Unspecified
Munter et al., 2015 [34]	Physicians	Night shifts (unspecified)	Indoor
Kwon et al., 2015 [35]	Factory workers	A night shift from 10 p.m. to the next morning at 6.a.m. at least four times per month on average or worked an average of at least 60 h per month during the night shift.	Indoor
Kantermann et al., 2014 [25]	Factory workers	Slow counterclockwise shifts: 6 days night (22–6 h), one off and 6 days morning (6–14 h), one off and 6 days late (14–22), one day off	Unspecified
Jeong et al., 2014 [26]	Unspecified—National survey	Those who worked in the afternoon (2 p.m. to midnight), at night (from 9 p.m. to 8 a.m. the following day), in regular rotation of shifts between day shifts and the night shifts, in 24 h shifts, in segmented shifts (working more than two shifts a day), and in irregular shifts	Unspecified
Kim et al., 2013 [27]	Unspecified—National survey	The following categories: (1) evening (14:00–24:00), (2) night (21:00–08:00), (3) regular shift time (day and night or regular 24 h), or (4) irregular shift time (includes two times or more in a day)	Unspecified

**Table 3:** Scoring of Cambridge Quality Checklists on included studies.

Authors, Year	Checklist for Correlates (0–5)	Checklist for Risk Factors (1–3)	Checklist for Causal Risk Factors (1–7)	Total Score (2–15)
Yang et al., 2021 [24]	2	1	2	5
Park et al., 2020 [28]	3	1	2	6
Rizza et al., 2020 [29]	2	1	2	5
Lee et al., 2020 [12]	3	1	5	9
Park et al., 2019 [30]	2	1	2	5
Alefishat et al., 2016 [31]	2	1	2	5
Erden et al., 2016 [32]	2	1	2	5
Romano et al., 2015 [33]	2	1	2	5
Munter et al., 2015 [34]	2	1	2	5
Kwon et al., 2015 [35]	2	1	2	5
Kantermann et al., 2014 [25]	2	1	2	5
Jeong et al., 2014 [26]	2	1	2	5
Kim et al., 2013 [27]	2	1	2	5

Checklist for correlates (adequate sampling method, adequate response rates, adequate sample size, good measure of correlate, good measure of outcome; one point each). Checklist for risk factors (cross-sectional data = 1; retrospective data = 2; prospective data = 3). Causal risk factor (study without a comparison group, no analysis of change = 1; inadequately controlled study, no analysis of change = 2: study without a comparison group with analysis of change = 3; inadequately controlled study with analysis of change = 4; controlled non-experimental study, no analysis of change = 5; controlled non-experimental study, with analysis of change = 6; randomized experiment targeting a risk factor = 7).

## Data Availability

Not applicable.
